# Characterisation of the Chemical Composition and Structural Features of Novel Antimicrobial Nanoparticles

**DOI:** 10.3390/nano7070152

**Published:** 2017-06-23

**Authors:** Yuen-Ki Cheong, Jesus Calvo-Castro, Lena Ciric, Mohan Edirisinghe, Elaine Cloutman-Green, Upulitha Eranka Illangakoon, Qiang Kang, Suntharavathanan Mahalingam, Rupy Kaur Matharu, Rory M. Wilson, Guogang Ren

**Affiliations:** 1 School of Engineering and Technology, University of Hertfordshire, Hatfield AL10 9AB, UK; y.cheong2@herts.ac.uk; 2 School of Life and Medical Sciences, University of Hertfordshire, Hatfield AL10 9AB, UK; j.calvo-castro@herts.ac.uk; 3 Department of Civil, Environmental and Geomatics Engineering, University College London, London WC1E 7JE, UK; l.ciric@ucl.ac.uk; 4 Department of Mechanical Engineering, University College London, London WC1E 7JE, UK; m.edirisinghe@ucl.ac.uk (M.E.); upulitha.illangakoon.12@ucl.ac.uk (U.E.I.); suntharavathanan.mahalingam@ucl.ac.uk (S.M.); rupy.matharu.15@ucl.ac.uk (R.K.M.); 5 Department of Microbiology, Virology, and Infection Prevention Control, Great Ormond Street Hospital NHS Foundation Trust, London WCIN 3JH, UK; elaine.cloutman-green@gosh.nhs.uk; 6 Institute of Metal Research, Chinese Academy of Science, 72 Wenhua Road, Shenyang 110016, China; qkang@imr.ac.cn; 7 Materials Research Institute, Queen Mary University of London, London E1 4NS, UK; r.m.wilson@qmul.ac.uk

**Keywords:** antimicrobial, antiviral, antibacterial, nanoparticles, tungsten, carbide, Raman, XRD, SS-NMR, XPS

## Abstract

Three antimicrobial nanoparticle types (AMNP0, AMNP1, and AMNP2) produced using the Tesima^TM^ thermal plasma technology were investigated and their compositions were determined using a combination of analytical methods. Scanning electron micrographs provided the morphology of these particles with observed sizes ranging from 10 to 50 nm, whilst FTIR spectra confirmed the absence of polar bonds and organic impurities, and strong Raman active vibrational bands at ca. 1604 and 1311 cm^−1^ ascribed to C–C vibrational motions were observed. Carbon signals that resonated at δ_C_ 126 ppm in the solid state NMR spectra confirmed that sp^2^ hybridised carbons were present in high concentration in two of the nanoparticle types (AMNP1 and AMNP2). X-ray powder diffraction suggested that AMNP0 contains single phase Tungsten carbide (WC) in a high state of purity and multiple phases of WC/WC_1-x_ were identified in both AMNP1 and AMNP2. Finally, X-ray photoelectron spectral (XPS) analyses revealed and quantified the elemental ratios in these composite formulations.

## 1. Introduction

Nanoparticles (NP) have been extensively investigated in biomedical applications ranging from biomaterials, diagnostics, to therapeutic treatments for cancers and other related diseases [1,2,3,4,5,6,7,8,9,10]. Research into the use of engineered bulk size materials (i.e., stainless steels) [11,12,13], micro- [14,15], and nano- [1,16] sized materials (i.e., metal and metal oxides) against a spectrum of bacteria and viruses have been a popular field, especially since the SARS (Severe acute respiratory syndrome) outbreak in 2003. The continuation of growing numbers of infectious diseases in hospitals, [17] propagation of pathogens, and their resistance against conventional antibiotics [18] have significantly raised global concern. Therefore using nanomaterials as antibacterials complementary to antibiotics is highly promising and is gaining a large interest, as they may fill the gaps where antibiotics frequently fail [19]. 

The activity of a widening spectrum of engineered metal/metal-oxide nanoparticles which can counter a specific range of oral pathogens associated with *peri-implantitis* has been investigated [20]. The findings assisted in the development of novel and innovative antimicrobial agents in an era of ever-increasing antimicrobial resistance. The potential of using nanoparticles as antimicrobial agents raised concerns about the possibility of nanoparticle toxicity on the central nervous system (CNS) [21,22,23]. In vivo investigation using hippocampus cells in young rats suggested an interactive connection between nano ZnO/Ag/CuO and the effectiveness of learning ability and the flexibility of cognition [21,24,25,26]. Nevertheless, incremental nano-dosages increased the excitability in rat CA1 pyramidal neurons and confirmed a safety threshold of 0.05 wt % of injection suspension, which has provided an insight regarding the relative toxicity concern over the use of nanoparticles in biomedical engineering [27,28,29]. These findings proved the novelty of antimicrobial nanoparticles (AMNP) in a cost-effective fashion for inhibiting microbial growth, which may bring major changes in policy and new regulations on the wider uses of nanoparticles in biomedical healthcare [7,16,30]. 

AMNP derivatives were synthesized in a previous work with a stable efficacy (99.99% killing rates) in order to counteract a range of super bugs (i.e., *E. coli*, MRSA) and a range of viruses (SARS, H5N1, and Noroviruses) [30,31]. As well as the accumulative biological results obtained from AMNP powder suspensions, AMNP doped polymer fibres produced using pressurized gyration [32] were recently found to inhibit the growth of *P. aeruginosa*, a Gram-negative bacterial species that is commonly found in hospitals [33].

Although, AMNP nanoparticles exhibit antimicrobial functions, their chemical and particle characterisations are not documented. Furthermore, how they interact with microbes has not been directly investigated. Most known antibacterial nanomaterials interact electrostatically with the bacterial membrane causing membrane disruption [19,34]. Consequently, free radicals are produced and instigate secondary membrane damage causing protein malfunction and DNA destruction [3]. Other antimicrobial nanomaterials such as nitric oxide matrices involve photoactivation with RNS (Reactive oxygen and nitrogen species) [35] or polycationic nanomaterials induce signal secretion to promote programmed cell death [36]. 

In this paper, we report chemical analyses obtained from these AMNP series (AMNP0, AMNP1, and AMNP2) using a range of techniques including FTIR/Raman spectroscopy, Solid state Carbon-13 Nuclear Magnetic Resonance spectroscopy, Powder X-ray Diffraction and X-ray Photoelectron Spectroscopy [37]. Through the chemical investigation, we found that C, W, Ag, Cu, and O are the main elements contained in these AMNP formulations, and we also identified several phases present in these series (i.e., WC, WC_1−x_, CuO). By understanding the lattice structures and chemistry of these antimicrobial nanoparticle composites, we hope to identify biological surface-interactions between these ultra-small particles and the microbes. This will help in studying the reaction mechanisms involved in target bacterium or viruses, and will also help to design and create custom-made antimicrobial formulations in the future.

## 2. Results and Discussion

### 2.1. Particle Characteristics 

**Figure 1:**
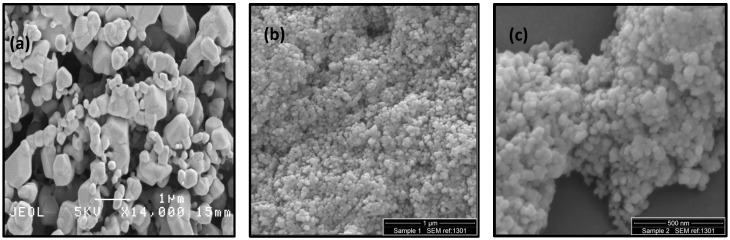
SEM images of (**a**) AMNP0; (**b**) AMNP1 and (**c**) AMNP2. AMNP denotes as Antimicrobial Nanoparticles.

The surface morphologies of the AMNP derivatives were studied using SEM. Figure 1a–c show the representative SEM images of AMNP0, AMNP1, and AMNP2, respectively. As shown in Figure 1a, the SEM image of the examined AMNP0 particles indicates that the sample was quite uniformly distributed. Although a large differentiation in their particle sizes were measured (50–500 nm), we were able to observe the apparent hexagonal units’ presence in these particles, which were later confirmed to be the P$\bar{6}$m2 tungsten carbide in our XRD study. In contrast, both SEM images of AMNP1 (Figure 1b) and AMNP2 (Figure 1c) showed a significant decrease in their particle sizes (10–30 nm). Both AMNP1 and AMNP2 were found to be heavily agglomerated and highly charged due to the nonconductive nature (i.e., non-metallic) of the samples. As a consequence, capturing good SEM images at higher resolution using accelerating voltage was difficult. 

In addition to the SEM analysis, EDX measurements were also performed with the aim of identifying possible elements present in these samples, and the results are presented and discussed in the atomic analysis section. 

### 2.2. Chemical Characteristics

**Figure 2:**
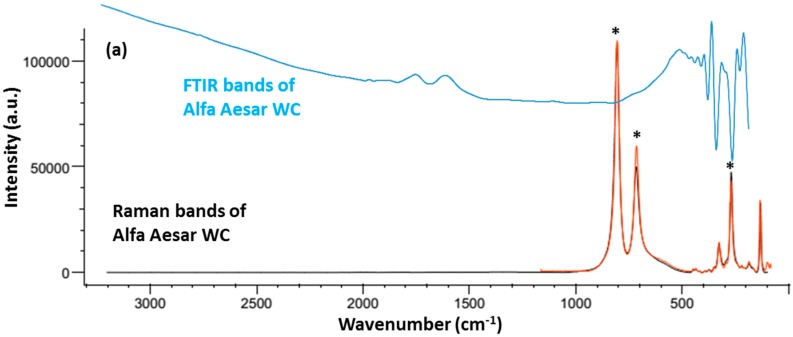

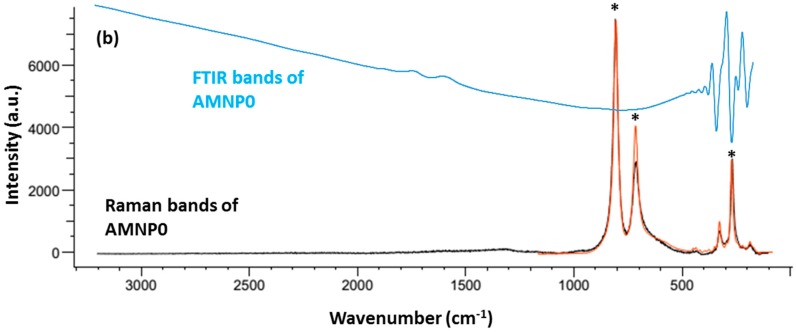
Raman and FTIR spectra of (**a**) Alfa Aesar tungsten carbide WC and (**b**) AMNP0. Spectra in red show reference Raman bands associate with related tungsten compounds. Symbols * denote the optimal vibrational Raman peaks observed at 803, 715 and 265 cm^−1^.

**Figure 3:**
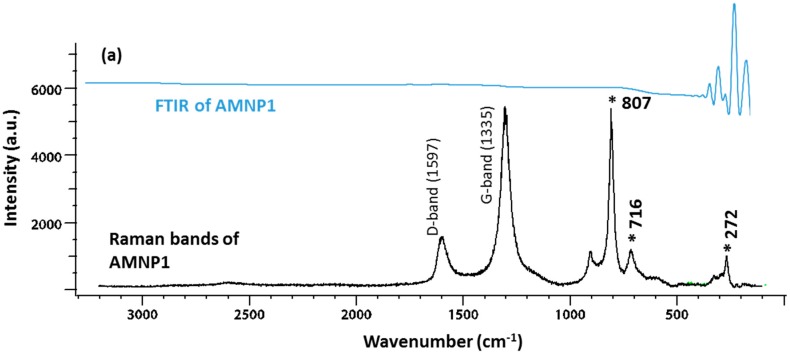

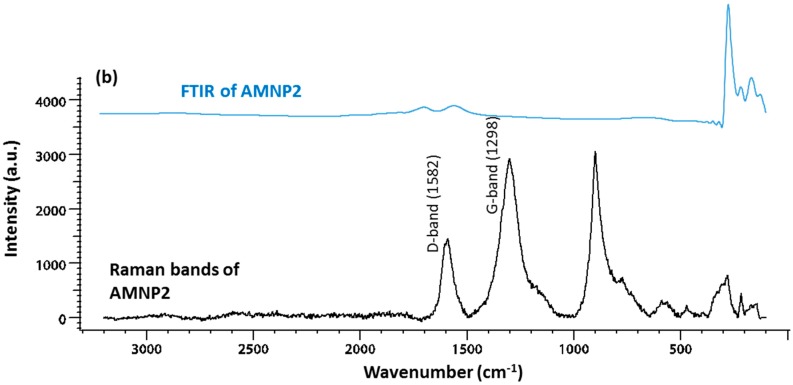
Raman and FTIR spectra of (**a**) AMNP1 and (**b**) AMNP2.

FTIR and Raman spectroscopy were used to identify the types of components (organic/inorganic/polymer) that may be contained in these nanoparticles. Raman and FTIR spectroscopies provided complimentary analyses to characterise the presence of intramolecular bonding in these composites. Four samples were investigated; this included the commercially available WC (<1 μm) from Alfa Aesear^®^ (Lancashire, UK), AMNP0, AMNP1, and AMNP2. Figure 2a shows the vibration spectra of Alfa Aesar WC in both FTIR (top) and Raman (bottom) modes, while Figure 2b shows the two spectra obtained from AMNP0. Similarly Figure 3a,b showed both FTIR and Raman vibration spectra of the AMNP1 and AMNP2 powder samples. The FTIR spectra of all four samples (top spectrum of Figure 2a,b and Figure 3a,b) showed only symmetrical stretches near the low frequency fingerprint region (500–1000 cm^−1^). No distinctive asymmetrical vibration stretches were observed in any of these samples, which suggested that these samples were clean of organic contaminants (i.e., C=O, CH, H_2_O). 

Upon detailed analysis of the Raman spectra acquired, additional composition information was obtained in line with the characteristic spectral profiles. In this regard, Raman active vibrational bands at ca. 265, 715, and 803 cm^−1^ observed in the AMNP0 spectrum (Figure 2b) coincide with those for the commercial WC sample [38] (Figure 2a), associated to stretching vibrational motions of the WC triple bond. In turn, the Raman spectra for AMNP1 and AMNP2 (Figure 3a,b) were observed to exhibit significant differences with respect to those of the commercial WC and AMNP0. In summary, clearly defined vibrational bands centred at ca. 1335/1298 and 1582/1597 cm^−1^ were observed for AMNP1/AMNP2, respectively, with the Raman active peaks at lower frequency associated with the so-called D-band characteristic of the A_1g_ mode of diamond type carbon with sp^3^ hybridisation. Thus, the vibrational bands at higher frequency were observed to be in agreement with the G vibrational band (E_2g_) characteristic of sp^2^ hybridised graphitic carbon [38,39]. The relative intensity of the D and G vibrational bands indicates a greater degree of graphitisation in AMNP2 [40]. In addition, Raman active vibrational bands at ca. 807, 716, and 272 cm^−1^ in the spectrum of AMNP1 (Figure 3a) are consistent with the presence of WC (vide supra). In fact, these active Raman bands may also be present in AMNP2 (Figure 3b), only it was swarmed by overlapping with other active signals due to the high complexity of the samples. 

**Figure 4:**
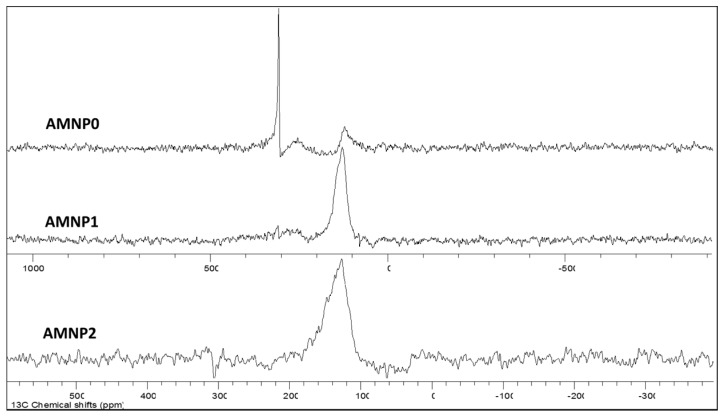
NMR spectra of AMNP0, AMNP1, and AMNP2.

^13^C-NMR spectroscopy is probably one of the best techniques to analyse and study carbon environments. We were unable to obtain a ^13^C signal from the commercial WC, however, this may be due to the paramagnetic effect caused by the presence of impurities in the commercial sample which was detected later from the XRD analysis. At present, there is insufficient NMR reports relating to metal carbides, whereas here we report the first ^13^C-NMR resonance observed in a WC sample. The lack of NMR study in metal carbides is due to the lack of aliphatic carbon (CH) present in carbide ions and also the attribution of the sp hybridised carbon, which are known to largely reduce the sensitivity of detection. With the chosen parameters (10 s relaxation delay) applied to these NMR experiments, we were able to observed three active carbon chemical signals resonating at δ_C_ 123, 254, and 307 ppm (top spectrum in Figure 4) in the AMNP0 sample. The one that resonated at δ_C_ 123 ppm is typically associated with aromatic/graphitic/charcoal type of carbon. Whereas the other two deshielded resonances at δ_C_ 254 and 307 ppm indicated carbon environments that are less electronegative, which match the chemical property of a carbide type carbon [41]. In contrast, the signal observed at δ_C_ 254 ppm was indeed comparable to the NMR shifts observed in Scandium carbide Sc_2_C_2_ (δ_C_ 253 ppm) and Yttrium carbide Y_2_C_2_ (δ_C_ 257 ppm) reported by Yamazaki et al. [41] and Zhang et al. [42]. An exceedingly rare ^13^C-NMR resonance published by Buss et al. [43] observed a carbon resonance exhibited at δ_C_ 546 ppm from a novel molybdenum carbide complex, thus the unusually sharp signal observed at δ_C_ 307 ppm could also be interpreted as a metal carbide resonance. 

The NMR investigations of AMNP1 and AMNP2 (Figure 4) are straight forward, and the only observable ^13^C chemical shifts resonated at 126 ppm are again associated with the presence of graphene/graphite types of carbons in both samples. Such high aromatic carbon contents found in both AMNP1 and AMNP2 NMR studies support the hypothesis that was drawn from the previous Raman analyses.

### 2.3. Phase Identification

**Figure 5:**
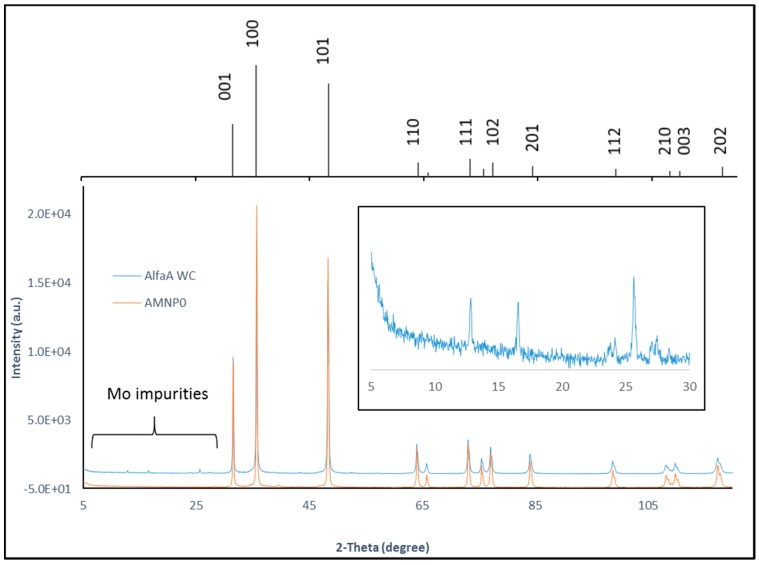
XRD patterns of AMNP0 and Alfa Aesar WC (insert diagram shows the presence of Mo impurities in the commercial sample). Top XRD stick pattern shows hexagonal P$\bar{6}$m2 WC obtained from the International Centre Diffraction Data service (ICDD 00-051-0939).

Powder X-ray analyses of the commercial WC and AMNP0 samples have unambiguously confirmed that these two samples contain single phase hexagonal WC, known as Qusongite [44]. Figure 5 shows the resulting XRD patterns of the commercial WC and AMNP0 samples against the reference supported by the International Centre of Diffraction Data (ICDD 00-051-0939) is shown as stick diagram in Figure 5). XRD analyses also identified 0.3–0.5% of the impurities as monoclinic MoO_3_ and hexagonal MoS_2_ (see insert diagram in Figure 5) in the commercial WC sample [45,46,47], these patterns match those provided by the database service (ICDD 04-004-5723 and ICDD 01-073-1544).

**Figure 6:**
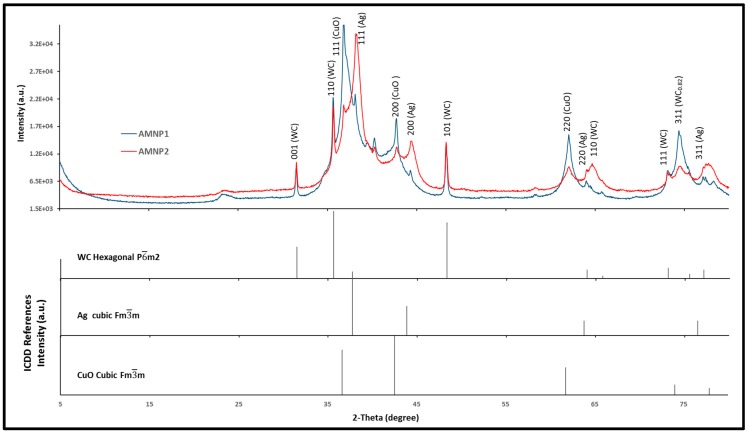
XRD patterns of AMNP1 in blue and AMNP2 in red. Stick diagrams on the bottom show XRD references extracted from the ICDD service.

As expected, a higher degree of broadening effects were observed in the diffractograms obtained from AMNP1 and AMNP2 (Figure 6), which indicated decreasing particle sizes in these samples. Such broadening may also be attributed to the multi-metallic elements present in high carbon content samples. The absence of the graphitic peak at 26° 2θ implies that the carbon is likely to be mainly amorphous. 

As shown in Figure 6 (blue diffractogram), multiple phase tungsten carbides were identified in AMNP1; this includes hexagonal WC (P$\bar{6}$m2) and two carbon deficient ones, W_2_C/WC_0.5_ and cubic WC_0.82_ (Table 1) [48,49]. As well as tungsten carbide derivatives, some W and Ag in cubic forms were also found in both AMNP1 and AMNP2, and the selective patterns extracted from the ICDD references are represented as stick diagrams on the bottom of Figure 6 [50,51]. As indicated, a trace amount of cubic CuO was also detected in the AMNP2 sample [52]. However, in this case, the XRD results obtained from AMNP1 and AMNP2 were not used to estimate the atomic arrangement and to quantify the elemental ratios in these powders due to the heavy broadening effects observed in these analyses. It should be noted that the XRD chemical analysis does not reveal all the details of small intermetallic powder samples (see our discussion in Section 2.4).

### 2.4. Atomic and Chemical State Analyses

**Figure 7:**
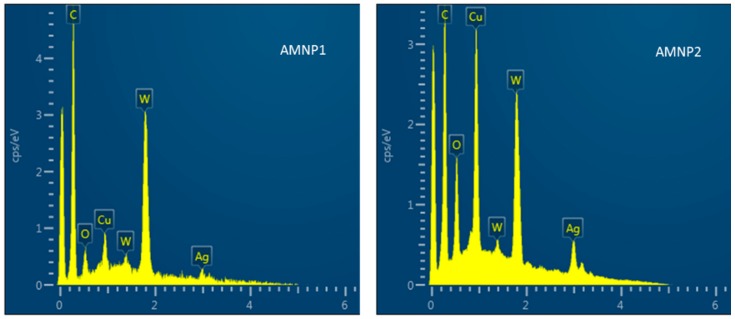
EDX spectra of AMNP1 and AMNP2.

Preliminary elemental analyses were performed using Energy dispersive X-ray spectroscopy, which was equipped with the Emission field SEM. Multiple point selective analyses of AMNP1 and AMNP2 (Figure 7) using EDX confirmed the presence of all of the elements (i.e., C, W, Ag, and Cu) found in previous Raman, NMR, and XRD analyses. 

**Figure 8:**
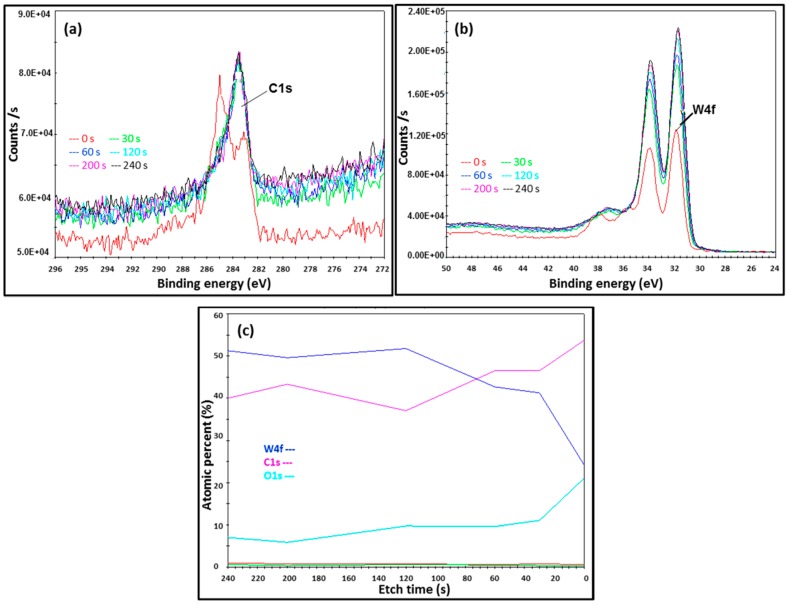
X-ray Photoelectron spectral Energy profiles of AMNP0 shows (**a**) the C1s binding energies and (**b**) the corresponding W4f binding energies; (**c**) shows the XPS atomic ratio analysis of AMNP0.

**Figure 9:**
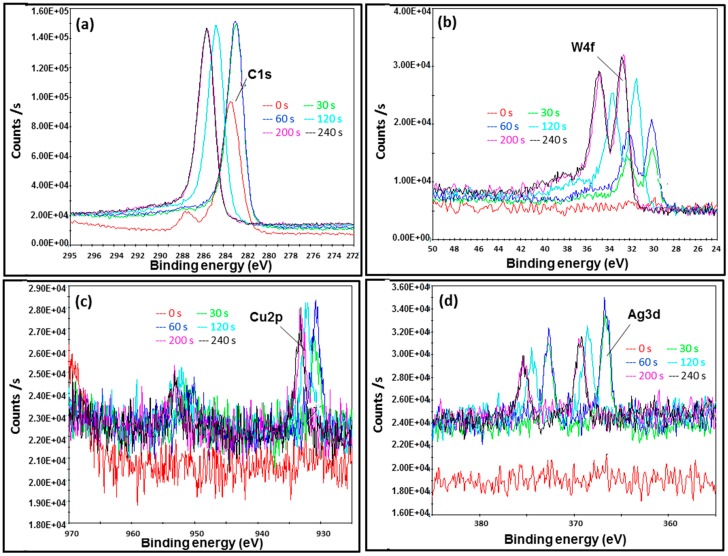
XPS Energy profiles of AMNP1 showing (**a**) binding energies of the corresponding C1s orbital; (**b**) the W4f; (**c**) the Ag3d and (**d**) the Cu2p, respectively.

**Figure 10:**
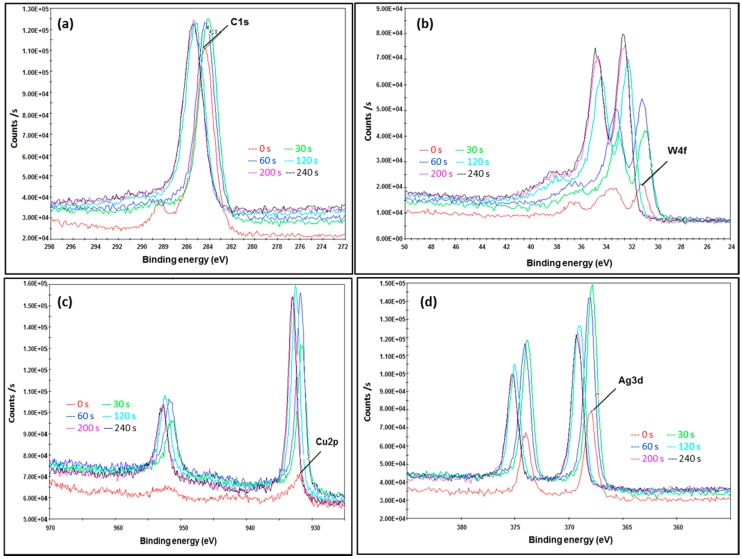
XPS Energy profiles of AMNP2 showing (**a**) the binding energies of the corresponding C1s orbital; (**b**) the W4f; (**c**) the Ag3d and (**d**) the Cu2p, respectively.

In order to investigate surface composition in the AMNP series and to quantify their elemental ratio, XPS analyses were performed. Figure 8, Figure 9 and Figure 10 show selected XPS energy profiles (C1s, W4f, Cu2p, and Ag3d) measured at different etch times (0, 30, 60, 120, 200, and 240 s) for the samples of AMNP0, AMNP1, and AMNP2 respectively. 

For AMNP0 (Figure 8), two binding energies at 285 and 283 eV (indicated in red in Figure 8a) were initially measured in the C1s spectrum at the 0 s etch time. C1s binding energies were then subsequently stabilised from etch time 30 s onwards. As expected, AMNP0 gave only singlets at 283.4 eV in the C1s spectrum, which is ascribed with the metal carbide energy state [39]. Figure 8b shows the XPS W4f spectrum acquired from AMNP0, consistent binding energies (eV) at 32, 34 (4f_7/2_ and 4f_5/2_ doublet), and 37 (5p_3/2_) were measured throughout, and these signals again indicated the presence of the carbidic bonding associated with WC in the AMNP0 [53].

Figure 9 and Figure 10 show the XPS energy profiles measured in AMNP1 and AMNP2. It is worth noting that the initial asymmetric peak at 284 eV with a tailed binding energy of 288 eV was found in both C1s spectra (Figure 9a and Figure 10a) in AMNP1 and AMNP2. These are associated with the common adventitious carbon contaminant (according to Thermo Scientific instruments application note). These peaks were subsequently removed by applying Argon clusters and were not observed at the 30 s etch time and onwards. Multiple binding energies (satellite features) were measured between 183 and 285 eV in the C1s spectra (Figure 9a and Figure 10a) indicating that both AMNP1 and AMNP2 have a high concentration of sp^2^ carbons [54,55]. This feature is also supported by the broad and asymmetric tail towards high eV, hence, if the sample contains a high sp^3^ concentration, the C1s peak will appear to be more symmetrical. Again the high content of graphitic carbon present in both AMNP1 and AMNP2 samples measured in the XPS analyses agrees with the results obtained from the corresponding Raman and NMR data reported earlier in Section 3.2.

As shown in Figure 9b, several binding energies were measured at the W4f core in AMNP1. They all appeared as asymmetric doublets at 30/32 eV, 31.5/33.5 eV, and 32.5/35 eV, and these three sets of doublets are believed to be associated with the multiple phases of W identified in the XRD results (Table 1). Metal Tungsten (W) has a reference XPS binding energy of 31.6/33.5 (doublet) and this W4f_7/2_/W4f_5/2_ feature in lost as the transition state of W changes to other W compounds (i.e., W_2_C/WC), hence eV shifts to higher energy are observed in the cases of both AMNP1 (Figure 9b) and AMNP2 (Figure 10b) [53]. Again, two sets of doublets in the W4f spectrum for AMNP2 (Figure 10b) were measured at 32/33.2 eV and 32/34.2 eV, which suggest different phases of the W compounds (i.e., W and WC) are present in AMNP2. 

Although Cu/CuO were not detected in AMNP1 during the XRD analysis, it was perhaps embedded beneath the particle surface. XPS analysis had clearly picked up a trace of Cu as observed in the Cu2p spectrum (Figure 9c), in which the binding energy was measured at 932 ± 1 eV as the etch level increased. Cu2p content was later quantified as 0.5% (Table 2) relative to the entire AMNP1 sample. Similar to the W4f core, significant split spin-orbit doublets (∆ = 19.75 eV) [53] were found in both XPS Cu2p spectra for AMNP1 (Figure 9c) and AMNP2 (Figure 10c). In contrast, the Cu content (CuO as identified from the XRD analysis) measured at 932.8/952.6 eV in AMNP2 is substantially higher than those found in AMNP1. 

Similarly, a significant amount of Ag in AMNP2 was detected when compared to AMNP1, and Figure 9d and Figure 10d represent the Ag3d spectra for AMNP1 and AMNP2, respectively. All of the Ag3d regions for both AMNP1 (368/374 eV) and AMNP2 (368/374 eV) samples, as expected, appeared as asymmetric doublets and have well separated spin-orbit components (∆ = 6.0 eV). Small shifts observed in these binding energies, particularly in AMNP1, may have been associated with the presence of oxides. Finally, the atomic ratio variation between C, O, W, Ag, and Cu were investigated. The atomic ratio of samples AMNP0, AMNP1, and AMNP2 were also measured during the XPS analyses at etch intervals of 0, 30, 60, 120, 200, and 240 s, and Figure 8c shows an illustrated sample of an atomic ratio diagram for the AMNP0. In summary, the overall atomic ratios were found and the average values were calculated using the data obtained from 30, 60, 120, 200, and 240 s intervals, as the sample surfaces at these intervals were stabilised under the conditions applied. The average atomic ratios for all three samples are given in Table 2.

Both Cu and Ag NP preparations, which are constituents of AMNP1 and AMNP2, have previously been shown to have good antimicrobial properties [56,57,58] and it is the presence of these compounds that is most likely to cause the antimicrobial effect seen in a previous study using these AMNPs incorporated into polymer mesh-like filters [33]. However, the exact content of these elements are not easy to determine in highly complex and heavily agglomerated nanopowders prepared using bulk forming methods.

## 3. Materials and Methods 

### 3.1. Materials

Commercial Tungsten Carbide (>99.5%) with particle sizes <1 μm was purchased from Alfa Asear (Lancashire, UK) and was used as a reference for a parallel study in this research. AMNP0, AMNP1, and AMNP2 were previously prepared by Qinetiq Nanomaterials^®^ using patented Tesima^TM^ thermal plasma technology (Farnborough, UK), the generic details of this production method are reported elsewhere [1,31]. All materials were used as received unless indicated otherwise. 

### 3.2. Scanning Electron Microscopy 

AMNP0, AMNP1, and AMNP2 particles were assessed using Scanning Electron Microscopy (SEM). AMNP0 (3 mg) was secured onto a carbon based adhesive substrate and positioned on a specimen stage, while powder samples (3 mg) of AMNP1 and AMNP2 were dispersed in 10 mL of ethanol prior analysis. From each dispersion, 10 µL was placed on separate SEM pin stubs and left to air dry. The samples were sputter-coated with 20 nm of gold for 180 s under argon using a Quorum Q150T Turbo-Pumped Sputter Coater (Essex, UK). AMNP0 was imaged using a JEOL JSM-6301F instrument (Welwyn Garden City, UK), whilst AMNP1 and AMNP2 were imaged using a Quanta 200 FEG ESEM (OR, USA). All images were collected with an accelerating voltage of 5 kV.

### 3.3. Fourier Transform Infrared and Raman Spectroscopy 

Infrared spectra were acquired using a PerkinElmer Frontier FT-IR/FIR spectrometer (Coventry, UK) equipped with an Attenuated Total Reflectance (ATR) accessory. Powder samples were loaded directly onto the diamond crystal stage and secured by a compressor rod. Blanks were performed prior to each sample submission, and all data were acquired at a resolution of 64 cm^−1^ using the built-in software ‘IRWinLab’ and 32 scans were collected.

Raman spectra were obtained by utilising a Renishaw InVia Raman microscope (Gloucestershire, UK) and associated WiRE 3.4 software supplied by the manufacturer. All measurements were performed by means of the 785 nm excitation wavelength and a 2 mW power laser. Powder samples were presented on a microscope slide with an approximate examined area of 20 × 20 µm^2^ and each measurement was taken after an average of 20 scans. The data were further analysed using the BioRed^®^ (Philadelphia, PA, USA) program and all visible Raman shifts were studied against the references supported by the database within the program.

### 3.4. Solid State Nuclear Magnetic Resonance Spectroscopy

Carbon-13 analyses were recorded at 100.6 MHz using a Bruker Avance III NMR spectrometer (Coventry, UK) equipped with a magic-angle spinning probe, in which the samples (~250 mg) were loaded in a 5 mm rotor (o.d.). Data were obtained using cross-polarisation with a 2 s recycle delay, 3 millisecond contact time, at ambient probe temperature (~25 °C), and at a sample spin-rate of 10 kHz. Between 1000 and 1600 repetitions were accumulated. Spectral referencing was with respect to an external sample of neat tetramethylsilane (carried out by setting the high-frequency signal from adamantine to 38.5 ppm). The ^13^C resonances apparent in the spectra represent the numbers of different chemical carbon environments emitted at specific frequencies and are measured as chemical shifts (δ) in ‘part per million’ (ppm). Data were analysed and processed using both Bruker Topspin (Coventry, UK) and Mestrec Nova (Santiago de Compostela, Spain).

### 3.5. X-ray Powder Diffraction 

Multiple XRD analyses were performed on each sample using different instruments and methods, and the selected XRD results of commercial WC and AMNP0 were acquired on a Panalytical X’Pert Pro diffractometer (Panalytical, Almelo, The Netherlands). This instrument was equipped with an X’Celerator solid state detector where Cu Kα was used as the radiation source with 0.25° divergence slits. The samples were mounted on zero background silicon single crystal substrates. Data were collected from 5 to 120° 2θ in steps of 0.033 degrees and a counting time of 200 s equivalent was used at each point. Whereas the selected XRD results of AMNP1 and AMNP2 were acquired from a Bruker D8 Advance X-ray diffractometer (Bruker AXS, Karlsruhe, Germany) using Cu Kα radiation. Data were collected from 5 to 80° 2θ in steps of 0.04° and a counting time of 1 s equivalent was used at each point. All four data sets were analysed using the HighScore program (Panalytical B.V.2012 version 3.0.5, Almelo, The Netherlands) and all visible XRD patterns were supported by matching ICDD references. 

### 3.6. Energy Dispersive X-ray Spectroscopy

Energy dispersive X-ray spectra of AMNP1 and AMNP2 were obtained using a JEOL field emission SEM JSM-7610F (Welwyn Garden City, UK) equipped with an Oxford Instruments (Oxfordshire, UK) 150 mm^2^ XMax^N^ silicon drift detector for energy dispersive spectroscopy. Powder samples (2–3 mg) were secured on carbon adhesive substrates and were positioned on a Eucentric specimen stage (5 axes motor control) before submitting to the SEM chamber for analyses. Built-in JEOL control software was used for all acquisitions, data collections, and image processing. All data were collected with an accelerating voltage of 0.1–30 kV, a spot size of 70 × 50 mm, and at a working distance of 1–40 mm.

### 3.7. X-ray Photoelectron Spectroscopy

All powder samples (1–2 g) were moulded and pressed into disks (2 × 2 mm^2^) with a thickness of 1 mm. Each disk sample was mounted on a stage where XPS measurements were performed using a Thermo VG Scientific ESCALAB250 (Waltham, MA, USA) surface analysis system equipped with a 3 kV Argon ion sputtering device operated at 500 eV (beam 2 μA, current 6 × 10^−8^ mbar) and at a sputter etching rate of 0.02 nm/s. The spectrometer used a monochromatic AlK_α_ as the radiation source with photon energies at 1486.6 eV and a spot size of 500 μm. The pass energy of 50 eV and energy step size of 0.1 eV were used in all experiments. Binding energies were recorded, and data were collected and analysed using the built-in software.

## 4. Conclusions

A combination of analytical methods including SEM, IR/Raman vibrational spectroscopy, ^13^C-NMR, XRD, and XPS were used to investigate the structure and chemical components contained in three types of antimicrobial nanoparticles. 

AMNP0 contains 99.8% single phase WC. As can be seen from both the FTIR/Raman spectra and the XRD analyses, no observable contaminant was detected in AMNP0 when compared with micro-metre sized commercial WC. This suggested that the production method (Tesima^TM^ thermal plasma technology) used to manufacture these formulations have reliable quality control. To further support the chemical content found in AMNP0, XPS analyses have recorded C1s and W4f binding energies emitted by the carbidic bonding presence in AMNP0. XPS analysis also measured the atomic ratio of the C1s:W4f content as 42.7:47.3%. It is also worth noting that a carbidic type of carbon signal (δ_C_ 254/307 ppm) associated with WC was detected using solid state NMR and it is the first report that has been published regarding this finding. 

As for AMNP1 and AMNP2, these both contain similar atomic components (C, O, W, Cu, and Ag), but in different ratios. Different phases of tungsten components (W, WC, WC_1−x_) were detected in AMNP1 and AMNP2, which may explain how each formulation exhibits different antimicrobial functions against specific bacteria/viruses. The high metallic ratio in AMNP2 was found to be five times more than that found in AMNP1 (17% vs. 3%) and can impart potential antimicrobial functions. The majority of the high carbon content found in both AMNP1 and AMNP2 (confirmed by Raman, NMR, and XPS analyses) may have been designed to increase bio-compatibilities and to reduce cytotoxicity effects.

The chemistries of these nano-powders, especially AMNP1 and AMNP2 produced using the thermal plasma process, were highly complex. Further analyses using more advanced characterisation methods (i.e., TEM and XRF) may allow a more in-depth understanding of these AMNP powders. In this aspect, identifications of any active sites and intermetallic properties present in the particles will provide insights for AMNP modifications and so to develop customised antimicrobial agents for selective pathogens. Extensive in vitro studies of these AMNP derivatives are being investigated, accumulating and collecting various analytical results from these nanopowders will help to understand the biological-chemical interactions between the surfaces of these particles and different microbes.

## Figures and Tables

**Table 1:** Visible components found in AMNP1 and AMNP2.

AMNP1		AMNP2	
Phases Detected	Details	Phases Detected	Details
Tungsten carbide (WC)	Hexagonal, P$\bar{6}$m2	Tungsten carbide (WC)	Hexagonal, P$\bar{6}$m2
Tungsten carbide (WC_0.82_)	Cubic, Fm$\bar{3}$m		
Tungsten carbide (W_2_C)	orthorhombic, Pbcn	Copper oxide (CuO)	Cubic, Fm$\bar{3}$m
Tungsten (W)	β-W, cubic Pm$\bar{3}$n	Tungsten (W)	β-W, cubic Pm$\bar{3}$n
Silver (Ag)	cubic, Fm$\bar{3}$m	Silver (Ag)	cubic, Fm$\bar{3}$m

**Table 2:** XPS atomic percentage for AMNP0, AMNP1 and AMNP2.

	C	O	W	Ag	Cu
AMNP0	42.7%	8.7%	47.3%	0.5%	0.8%
AMNP1	94.0%	2.9%	2.1%	0.5%	0.5%
AMNP2	77.7%	5.5%	6.7%	4.7%	5.4%
